# PER-14, a β-lactamase variant with reduced sensitivity to avibactam and relebactam

**DOI:** 10.1128/aac.00378-26

**Published:** 2026-07-21

**Authors:** Melina Ruggiero, Melina García Alonso, Salvador I. Drusin, Roque Figueroa-Espinosa, Ivan Jackson Briceño Muñoz, Robert A. Bonomo, Gabriel Gutkind, José Di Conza, Diego M. Moreno, Pablo Power

**Affiliations:** 1 Universidad de Buenos Aires, Facultad de Farmacia y Bioquímica, Instituto de Investigaciones en Bacteriología y Virología Molecular (IBaViM) 62963, Buenos Aires, Argentina; 2 Consejo Nacional de Investigaciones Científicas y Técnicas (CONICET) 62873, Buenos Aires, Argentina; 3 IQUIR (Instituto de Química Rosario), Consejo Nacional de Investigaciones Científicas y Técnicas (CONICET), Universidad Nacional de Rosario 28237, Rosario, Argentina; 4 Facultad de Ciencias Bioquímicas y Farmacéuticas, Universidad Nacional de Rosario 63029, Rosario, Argentina; 5 Medical Service, Louis Stokes Cleveland VA Medical Center 20083, Cleveland, Ohio, USA; 6 GRECC, Louis Stokes Cleveland VA Medical Center 20083, Cleveland, Ohio, USA; 7 Department of Medicine, Case Western Reserve University School of Medicine 220786, Cleveland, Ohio, USA; 8 Department of Pharmacology, Case Western Reserve University School of Medicine 12304, Cleveland, Ohio, USA; 9 Department of Molecular Biology and Microbiology, Case Western Reserve University School of Medicine 196211, Cleveland, Ohio, USA; 10 Department of Biochemistry, Case Western Reserve University School of Medicine 12304, Cleveland, Ohio, USA; 11 Department of Proteomics and Bioinformatics, Case Western Reserve University School of Medicine 682976, Cleveland, Ohio, USA; 12 CWRU-Cleveland VAMC Center for Antimicrobial Resistance and Epidemiology (Case VA CARES) 2546 , Cleveland, Ohio, USA; University of Fribourg, Fribourg, Switzerland

**Keywords:** *Pararheinheimera*, ceftazidime/avibactam, ceftazidimase, ESBL, diazabicyclooctane, aztreonam/relebactam

## Abstract

PER-1-producing *Pseudomonas *and *Acinetobacter* and PER-2-producing *Enterobacterales*, respectively, are increasingly difficult to treat due to combined resistance to antibiotics like ceftazidime, cefiderocol, and ceftazidime/aztreonam-avibactam, among others. PER-14 differs from PER-2 by an S130T substitution. Through combined functional and *in silico* analyses, we demonstrated that PER-14 is resistant to avibactam and relebactam inhibition while retaining an extended-spectrum activity and the ability to hydrolyze cefiderocol, as the S130T substitution affects the proper binding of the DBOs.

## INTRODUCTION

The PER family of class A serine β-lactamases has been likely recruited from different environmental *Pararheinheimera* species (1). From the 18 recognized variants (http://bldb.eu/BLDB.php?prot=A#PER), PER-1 and PER-2 are the most frequently reported in *Pseudomonas*/*Acinetobacter* and *Enterobacterales*, respectively (2).

The diazabicyclooctane inhibitors (DBOs) avibactam (AVI) and relebactam (REL) are currently employed for the treatment of infections caused by multidrug-resistant gram-negative bacteria (3). Although AVI and REL exhibit potent activity against most serine-β-lactamases, PER β-lactamases are associated with resistance to ceftazidime-avibactam and aztreonam-avibactam (ATM-AVI) in *Pseudomonas* spp. and *Enterobacterales* (4–7). In previous studies, the efficacy of AVI against PER-2 was demonstrated to be lower compared to other class A β-lactamases (8, 9). Additionally, the co-production of NDM and PER β-lactamases is associated with decreased susceptibility or resistance to cefiderocol (FDC) in *Pseudomonas aeruginosa*, *Providencia rettgeri*, and *Acinetobacter baumannii* (5, 10, 11). PER-2 was demonstrated to hydrolyze FDC with catalytic efficiency values equivalent to NDM-1 (12). The INFORM global surveillance study (2012–2017) identified a *Raoultella ornithinolytica* isolate from Argentina with an ATM-AVI MIC of 16/4 μg/mL, carrying a novel PER variant assigned as PER-14 (4).

This study aims to provide a phenotypic and kinetic characterization of PER-14. *bla*_PER-14_ differs from *bla*_PER-2_ by a single nucleotide resulting in an S130T substitution, which was previously linked to increased AVI resistance in PER-4 (7, 13). In class A β-lactamases, S130 has an important role during acylation, as it participates in proton shuttling, substrate recognition, and inhibitor binding (14, 15).

The *bla*_PER-14_ and *bla*_PER-2/S130G_ genes were generated by site-directed mutagenesis from *bla*_PER-2_ using a PCR-based overlap extension protocol (16); the PER-2^S130G^ variant was designed to better assess the impact of substitutions at amino acids in position 130. *Escherichia coli* TOP10F′ was transformed with pUC57-kan constructions containing the cloned *bla*_PER_ genes, and the MICs were determined according to CLSI guidelines (17).

The MICs for tested antibiotics were similar for both PER-14- and PER-2-producing clones except for ATM, where the MIC decreased by four dilutions in the PER-14-producing clone (Table 1). AVI and REL failed to reduce MICs of CAZ or ATM in the PER-14-producing strain, although they were efficient in reducing the MICs by up to nine dilutions in the PER-2-producing clone. Sulbactam and clavulanic acid effectively reduced MICs for ampicillin (AM) and ATM, respectively, in both PER-2- and PER-14-producing clones (Table 1). For PER-2^S130G^, a similar MIC value was obtained only for AM. All cephalosporins and ATM exhibited considerably lower MIC values, indicating the inability of this variant to efficiently hydrolyze these substrates. Increased susceptibility to FDC was also observed in the PER-2^S130G^ variant.

**TABLE 1:** MIC values of β-lactam antibiotics and combinations with β-lactamase inhibitors for PER-14 and PER-2-producing clones^*a*^

*E. coli* TOP10F′		MIC (μg/mL)										
	AM	AMS	CF	CRO	CAZ	CAZ-AVI	FEP	FDC	ATM	ATM-AVI	ATM-REL	ATM-CLA
PER-2/pUC57-kan	>512	16/8	256	128	512	2/4	16	0.5	1,024	2/4	1/4	0.25/4
PER-14/pUC57-kan	256	64/32	256	64	128	128/4	8	0.5	64	64/4	32/4	0.25/4
PER-2^S130G^/pUC57-kan	512	16/8	8	0.25	16	4/4	0.5	0.03	8	2/4	8/4	0.5/4
pUC57-kan	4	4/2	4	0.06	0.25	0.25/4	0.06	0.03	0.25	0.25/4	0.25/4	0.25/4

^*a*^ AM, ampicillin; AMS, ampicillin-sulbactam; ATM, aztreonam; ATM-AVI, aztreonam-avibactam; ATM-CLA, aztreonam-clavulanate; ATM-REL, aztreonam-relebactam; CAZ, ceftazidime; CAZ-AVI, ceftazidime-avibactam; CF, cephalothin; CRO, ceftriaxone; FEP, cefepime; FDC, cefiderocol.

PER-14 and PER-2^S130G^ production and purification, as well as the evaluation of AVI and REL inhibition using 100 μM nitrocefin (NCF) as a reporter substrate, were performed as previously described (9). As the catalytic behavior of the enzymes remains reproducible, we used this feature, combined with assessment of protein purity by SDS-PAGE, as adequate criteria to estimate protein integrity and purity.

Kinetic data supported antimicrobial susceptibility behavior (Table 2). Catalytic efficiencies (*k*_cat_/*K_m_*) were comparable between PER-2 and PER-14 for all tested antibiotics. We previously demonstrated that PER-2 is inhibited by AVI with acylation rates reminiscent of values observed for class C and D β-lactamases (*k*_2_/*K* of ≅10^3^ M^−1^ s^−1^) but lower than other class A β-lactamases (*k*_2_/*K* of ≅10^4^ to 10^5^ M^−1^ s^−1^) (8, 9). When comparing the steady-state inhibition of PER-14 and PER-2 by DBOs (Table 3), the *K_i_*_app_ of AVI was 37-fold higher for PER-14, while the *k*_2_/*K* decreased by approximately 880-fold, indicating that in PER-14, both the binding and the acylation step are compromised. For REL, the *K_i_*_app_ was 200-fold higher for PER-14, while the *k*_2_/*K* was reduced by approximately 3,200-fold. The deacylation rate constant (*k*_off_) for the PER-2/AVI and PER-2/REL was equivalent (0.0004 and 0.0002 s^−1^, respectively) and comparable to other class A β-lactamases (8). We were unable to determine the *k*_off_ for PER-14, mainly because of very high *K*_*m*_ values (low affinity) and/or low *k*_2_/*K* constants (slow acylation rate).

**TABLE 2:** Steady-state kinetic parameters for PER-14, PER-2^S130G^, and PER-2 against β-lactam antibiotics^*e*^

Antibiotic	PER-14 (PER-2^S130T^)			PER-2^S130G^			PER-2^*c*^		
	*K_m_* (μM)	*k*_cat_ (s^−1^)	*k*_cat_/*K_m_* (μM^−1^·s^−1^)	*K_m_* (μM)	*k*_cat_ (s^−1^)	*k*_cat_/*K_m_* (μM^−1^·s^−1^)	*K_m_* (μM)	*k*_cat_ (s^−1^)	*k*_cat_/*K_m_* (μM^−1^·s^−1^)
AM	30 ± 2	54.0 ± 0.5	1.8 ± 0.1	3.13 ± 0.05	21.3 ± 0.3	6.8 ± 0.2	18 ± 1	53.0 ± 0.5	3.0 ± 0.2
CF^*a*^	9 ± 1	84 ± 2	9 ± 1	17 ± 1	0.96 ± 0.02	0.005 ± 0.006	12.7 ± 0.7	49.0 ± 0.8	3.9 ± 0.3
CRO^*b*^	28 ± 2	65 ± 1	2.4 ± 0.2	14.0 ± 0.7	0.61 ± 0.01	0.044 ± 0.003	51 ± 3	92 ± 3	1.8 ± 0.1
CAZ	126 ± 10	103 ± 4	0.82 ± 0.09	ND	ND	0.0085 ± 0.0002	105 ± 4	75 ± 1	0.71 ± 0.04
FDC	ND	ND	0.186 ± 0.005	ND	ND	0.00121 ± 0.00004	ND	ND	0.072 ± 0.002
FEP	19 ± 2	19.8 ± 0.7	1.1 ± 0.1	ND	ND	0.086 ± 0.002	40 ± 2	18.6 ± 0.6	0.47 ± 0.05
ATM^*a*^	1.20 ± 0.03	1.87 ± 0.08	1.57 ± 0.05	44 ± 7	5.106 ± 0.002	0.12 ± 0.02	2.4 ± 0.1	4.8 ± 0.6	1.98 ± 0.05
NCF^*d*^	12 ± 1	241 ± 7	20 ± 3	64 ± 6	689 ± 30	11 ± 2	32 ± 2	158 ± 3	5.0 ± 0.4

^*a*^ *K*_*m*_ values were determined as *K_i_* with nitrocefin as reporter substrate.

^*b*^ Results for PER-2 are for cefotaxime.

^*c*^ PER-2 kinetic parameters are from references 12 and 18.

^*d*^ Nitrocefin hydrolysis parameters for PER-14 were calculated using the model for inhibition by substrate from Verlaine et al. (19). PER-14 T130G shows a competitive inhibition by product.

^*e*^ AM, ampicillin; ATM, aztreonam; CAZ, ceftazidime; CF, cephalothin; CRO, ceftriaxone; FDC, cefiderocol; FEP, cefepime; NCF, nitrocefin; ND, not determined.

**TABLE 3:** Inhibition kinetic parameters for PER-14 and PER-2 against AVI and REL^*a*^

Kinetic parameters	PER-14 (PER-2^S130T^)	PER-2^*b*^
AVI		
*k_2_*/*K* (M^−1^·s^−1^)	2.5 ± 0.2	2,200 ± 100
*K_i_*_app_ (μM)	740 ± 40	20 ± 3
*k*_off_ (s^−1^)	ND	0.0004
REL		
*k*_2_/*K* (M^−1^·s^−1^)	3.5 ± 0.2	11,200 ± 600
*K_i_*_app_ (μM)	10,000 ± 700	5.0 ± 0.6
*k*_off_ (s^−1^)	ND	0.0002

^*a*^ ND, not determined (see text for details).

^*b*^ AVI values from reference 9, REL values from reference 20.

For PER-2^S130G^, similar *k*_cat_/*K_m_* values were only obtained for the hydrolysis of AM and NCF (Table 2), although a competitive inhibition by NCF product was observed (19). For the rest of the antibiotics, the *k*_cat_/*K_m_* decreased considerably by a factor higher than 10, mainly due to a decrease in the *k*_cat_. For FDC, the catalytic efficiency of this variant was up to 150 times lower than PER-2 and PER-14, in agreement with the observed decrease in the MIC. AVI and REL were also unable to efficiently inhibit the enzyme (Table 1), and since the enzyme cannot properly hydrolyze CAZ or ATM either, a combination of these inhibitors and antibiotics would be effective for PER-2^S130G^. These observations are consistent with isolates producing other class A β-lactamases (i.e., KPC-2 or CTX-M-15), where the S130G substitution confers AVI resistance, but the enzymes also lose the ability to hydrolyze cephalosporins (21, 22).

Molecular dynamics (MD) simulations and docking studies were performed to gain structural insight into the reduced susceptibility of PER-14 to DBO inhibitors, according to Drusin et al. (23). The experimental details are provided as Text S1 in the supplemental material.

**Fig 1:**
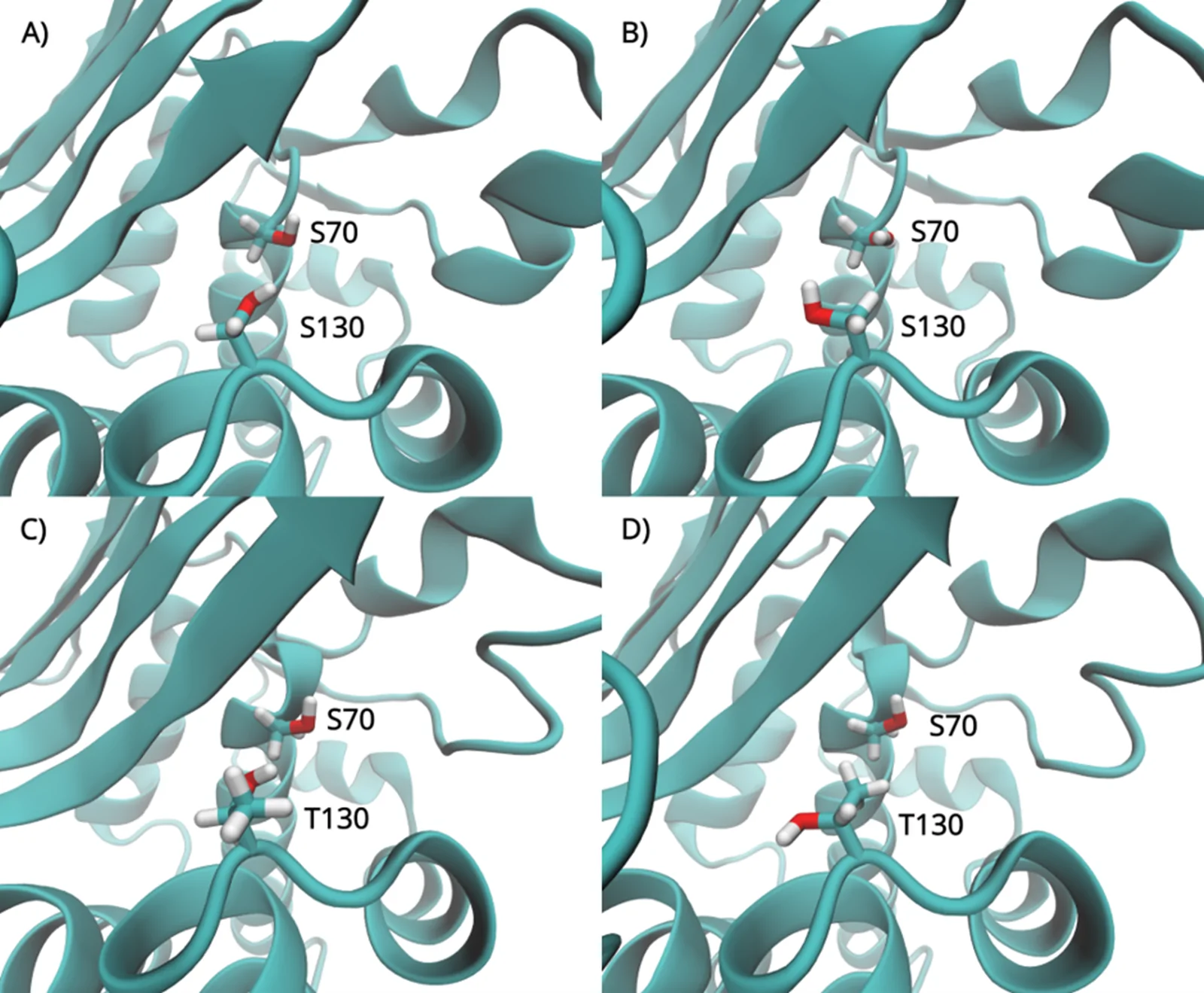
Representative orientations of residues 70 and 130 in PER-2 (**A and B**) and PER-14 (**C and D**) obtained from MD simulations.

The MD simulations revealed a marked difference between PER-2 and PER-14 in the conformational behavior of residue 130. In PER-2, S130 fluctuated between two preferred orientations: one in which the hydroxyl group points toward the inhibitor-binding region, as observed in the crystallographic structure, and another in which the side chain is rotated by ~120°, orienting the hydroxyl group away from the binding pocket (Fig. 1A and B). In contrast, in PER-14, T130 rapidly adopted and maintained a rotated conformation in which the hydroxyl group points away from the active site (Fig. 1C and D), and the methyl group of T130 becomes oriented toward the region usually occupied by the sulfate group of DBOs. This conformational rearrangement is mechanistically relevant because residue 130 participates in the hydrogen-bonding and proton-transfer network required for efficient acylation by DBO inhibitors (14, 15). Therefore, the outward orientation of the T130 hydroxyl group, together with the placement of its methyl group toward the inhibitor-binding region, may perturb the optimal pre-acylation geometry without preventing substrate binding. The time evolution of the side-chain dihedral angle of residue 130 further supports this interpretation, showing that PER-14 remains in the rotated state, whereas PER-2 samples both conformations (Fig. S1).

Based on this observation, molecular docking calculations of AVI and REL were performed to evaluate how this local rearrangement affects inhibitor recognition by both PER-2 (for comparison) and PER-14. Overall, as observed in Fig. S2, the two inhibitors adopt productive poses oriented toward the catalytic S70 while engaging with key residues involved in substrate recognition and catalysis, including S/T130, R220, K234, T235, and T237 (through electrostatic/polar interaction with the sulfonate group), and with residues Q69, N132, E166, and H170 (by polar interaction with the amide/piperidin-4-yl carbamoyl group), which is consistent with a productive pre-acylation complex, as previously reported in the structure of PER-2 acylated by AVI (PDB 6D3G) (9). However, the PER-14 conformation with the outward-facing T130 hydroxyl group yielded less favorable docking solutions for both AVI and REL compared with the initial PER-14 structure, showing a 0.70 and 0.74 kcal/mol decrease in predicted binding energy and a 52% and 7% reduction in the population of the selected productive poses, respectively (Fig. S3).

To experimentally assess whether these structural differences affected avibactam trapping, intact-protein MALDI-TOF analyses were performed after incubation of PER variants with AVI (Text S2; Fig. S4). PER-2 rapidly formed an adduct-compatible species under equimolar conditions (1:1), detectable within 5 min and persisting throughout incubation. In contrast, PER-14 showed no detectable adduct accumulation under equimolar conditions and only exhibited an adduct-compatible mass shift after prolonged incubation under large inhibitor excess (1:50). These findings support impaired acyl-enzyme accumulation in PER-14 despite residual inhibitor recognition.

Together, these results support a model in which the S130T substitution does not abolish DBO binding but shifts the local conformational preferences toward less productive pre-acylation complexes. Although AVI and REL retain overall binding orientations toward the catalytic S70 in PER-14, the rotated T130 side chain and the altered local electrostatic environment reduce optimal inhibitor positioning for efficient acylation. These structural effects are modest at the docking level but are dramatically amplified kinetically, as reflected by the marked reductions in *k*_₂_/*K* and increases in *K*_*i* app_, underscoring the critical role of precise ligand positioning in covalent inhibition of PER-type β-lactamases. In contrast, the S130G substitution likely causes a more severe disruption of the catalytic hydrogen-bonding network due to complete loss of the residue 130 side chain, consistent with the broader functional impairment observed for this mutant.

Consistent with observations in other class A β-lactamases, residue S130 plays a central role in DBO inhibition by acting as a proton donor during acylation (21, 22, 24) and contributing to the inhibitor’s recyclization during deacylation (25). In PER-14, the additional methyl moiety of T130 likely reduces hydroxyl group flexibility compared to serine and introduces local steric constraints that impair productive inhibitor acylation while preserving β-lactamase hydrolysis. A mechanistic model is proposed in Fig. S5.

In this work, we demonstrate that PER-14 is resistant to inhibition by AVI and REL while retaining significant ESBL activity and the ability to hydrolyze FDC. Notably, *bla*_PER-14_ differs from *bla*_PER-2_ by a single nucleotide substitution, supporting the hypothesis that PER-14 evolved from PER-2 rather than arising from direct recruitment from *Pararheinheimera* spp. This is consistent with the stepwise accumulation of mutations that drive β-lactamase evolution, whereby limited genetic changes can result in advantageous amino acid substitutions. In this case, such evolution has led to a DBO inhibitor-resistant β-lactamase phenotype while preserving the ESBL profile of PER-2.

These features confer clear clinical relevance to PER-14, particularly in the context of the increasing use of FDC-, AVI-, and REL-based regimens as therapeutic options, which may favor its selection and therefore compromise the clinical efficacy of CAZ–AVI or ATM–AVI therapies.

## SUPPLEMENTAL MATERIAL

Supplemental material aac.00378-26-s0001.docx Fig. S1 to S5.

ASM does not own the copyrights to Supplemental Material that may be linked to, or accessed through, an article. The authors have granted ASM a non-exclusive, world-wide license to publish the Supplemental Material files. Please contact the corresponding author directly for reuse.
