# Supplementary material for: PER-14, a β-lactamase variant with reduced sensitivity to avibactam and relebactam
Source: Antimicrob Agents Chemother. 2026 Jul 21;70(9):e00378-26. doi: 10.1128/aac.00378-26 (PMC13712949; doi:10.1128/aac.00378-26)
Supplement: Supplementary file 1 [file aac.00378-26-s0001.docx]

**Supplemental material**

**PER-14, a Novel β-Lactamase Variant with Reduced Susceptibility to Avibactam and Relebactam**

Melina Ruggiero, Melina García Alonso, Salvador I. Drusin, Roque Figueroa-Espinosa, Ivan Jackson Briceño Muñoz, Robert A. Bonomo, Gabriel Gutkind, José Di Conza, Diego M. Moreno, Pablo Power

**Text S1**

**Molecular dynamics:**

Molecular dynamics (MD) simulations were performed using the PMEMD software from the AMBER24 package (1). The structure for PER-2 was taken from PDB ID 4D2O (2) and the PER-14 mutation (S130T) was modelled using Tleap. Both proteins were submerged in a periodic truncated octahedral box of water molecules with 0.1 M Na^+^ and Cl^-^. Parameters used for the proteins and water molecules were ff14SB (3), and TIP3P, respectively; the thermostat and barostat used were Langevin and Monte Carlo, respectively. Each simulation protocol started with 10,000 steps energy minimization, followed by 10 ps thermalization from 0 to 300 K at constant volume and 20 ps equilibration at constant temperature/pressure, ending in 500 ns production at constant temperature/pressure.

**Molecular docking:**

Molecular docking simulations were performed using Autodock-GPU (4). One hundred docking runs per simulation were performed, clustered using a RMSD cut-off of 2 Å. Between all the docking poses produced by the simulation, the productive pose of each ligand was selected, defined as the one in which the distance between the β-lactam’s carbonyl and the S70’s hydroxyl groups atoms was <3.0 Å. For the proteins, the same starting structure as for the molecular dynamics simulations were used, and new conformations in which residues S130 or T130 were rotated 120°. For the ligands, the structures were taken from PubChem and processed by optimization of geometry and calculation of RESP charges using Gaussian 16 (5) at B3LYP/6-31G** level. The ligands were then processed using Autodock Tools to prepare them for docking.

**Text S2**

**MALDI-TOF MS Analysis of Enzyme-Inhibitor Complexes:**

Following purification, recombinant PER-2 and PER-14 β-lactamases underwent thrombin cleavage to remove their affinity tags. The resulting untagged proteins were transferred into PBS buffer. To study how the enzyme-inhibitor complex forms and persists over time, we incubated these samples with avibactam at room temperature across several time points (5, 30, and 60 min, and 24 h). For PER-2 assays, both components were mixed at an equimolar final concentration of 20 µM (1:1 ratio). In contrast, PER-14 was subjected to two distinct setups: an equimolar condition (20 µM each, 1:1) and an inhibitor-excess condition containing 20 µM enzyme and 1000 µM avibactam (1:50 molar ratio).

We monitored intact protein masses via MALDI-TOF mass spectrometry, utilizing a Microflex LT linear-mode instrument operated in positive polarity (Bruker Daltonics, Bremen, Germany). Sample crystallization relied on a sinapinic acid (SA) double-layer approach. Initially, we anchored a basal layer by applying 0.1 µL of ethanol-saturated SA directly onto the stainless-steel target plate, allowing it to air-dry. The protein-avibactam incubation mixtures were subsequently blended 1:1 (v/v) with the matrix solution—comprising 10 mg/mL SA dissolved in a 30:70 acetonitrile to 0.1% aqueous trifluoroacetic acid mixture. A 1 µL aliquot of this final analyte-matrix blend was then layered on top of the dried base and left to crystallize prior to spectral acquisition.

Mass spectra acquisition spanned an *m/z* window of 15,000 to 50,000 Da, pooling 800 laser shots per profile. Standard calibration was performed externally using Protein Calibration Standard II from Bruker Daltonics. To ensure reproducibility, each combination of incubation time and experimental condition was assayed in separate, independent duplicates. Data processing was conducted via flexAnalysis v3.4 software, followed by meticulous visual inspection to track the characteristic mass shifts that denote covalent enzyme-avibactam adduct formation.

**Figure S1.** Dihedral angle of the atoms C_CO_-C_α_-C_β_-O_γ_ in residue 130 in PER-2 (blue line) and PER-14 (orange line).

**Figure S2.** Productive docking conformations for PER-2 with avibactam (A) and relebactam (B), PER-2 with a rotated S130 residue with avibactam (C) and relebactam (D), PER-14 with avibactam (E) and relebactam (F), and PER-14 with a rotated T130 residue with avibactam (G) and relebactam (H). Protein carbon atoms are shown in cyan while ligand carbon atoms are shown in orange, oxygen atoms are shown in red, nitrogen atoms in blue, and sulfur atoms in yellow. Hydrogen atoms are omitted for clarity.

**Figure S3.** Docking simulations results for avibactam and relebactam in PER-2 and PER-14 in all conformations. PER-2* and PER-14* are the conformations in which the residues S130 and T130 are rotated 120° with the alcohol group oriented away from the binding pocket.

**Figure S4.** Intact-protein MALDI-TOF analysis of avibactam-induced mass shifts in PER enzymes. MALDI-TOF mass spectra of purified PER-2 and PER-14 following incubation with Avibactam at different time points to assess formation of enzyme-inhibitor adduct-compatible species. Left column: PER-2 incubated with avibactam at a 1:1 molar ratio (20 µM enzyme, 20 µM avibactam). Middle column: PER-14 incubated with avibactam at a 1:1 molar ratio (20 µM enzyme, 20 µM avibactam). Right column: PER-14 incubated with avibactam at a 1:50 molar ratio (20 µM enzyme, 1,000 µM avibactam). The first row shows apo-enzyme spectra (without inhibitor). Rows 2–5 correspond to incubation times of 5 min, 30 min, 60 min, and 24 h, respectively. Peak labels indicate the measured intact protein masses (Da). PER-2 showed a rapid mass increase compatible with formation of an avibactam-bound species under equimolar conditions, detectable within 5 min and persisting throughout incubation. In contrast, PER-14 showed no detectable adduct accumulation under equimolar conditions and only exhibited a comparable mass shift after prolonged incubation (24 h) under inhibitor excess. These results support markedly impaired avibactam trapping by PER-14 relative to PER-2.

**Figure S5:** Hypothetical mechanisms for acylation of PER-2 (A), PER-14 (B), and PER-2^S130G^ (C) by avibactam. Predicted hydrogen bonds and salt bridges are shown as dashed green and yellow lines, respectively; the covalent bond in the acyl-enzyme is represented by a bold line. Red arrows represent the proton transfer pathway during acylation: a bold arrow is associated with a high acylation rate (PER-2), regular line represents the lower rate (PER-14), and a dashed arrow indicates a predicted negligible acylation in PER-2^S130G^ mutant. See text for details.

**References:**

1.	Case DA, Aktulga HM, Belfon K, Cerutti DS, Cisneros GA, Cruzeiro VWD, Forouzesh N, Giese TJ, Götz AW, Gohlke H, Izadi S, Kasavajhala K, Kaymak MC, King E, Kurtzman T, Lee T-S, Li P, Liu J, Luchko T, Luo R, Manathunga M, Machado MR, Nguyen HM, O’Hearn KA, Onufriev A V, Pan F, Pantano S, Qi R, Rahnamoun A, Risheh A, Schott-Verdugo S, Shajan A, Swails J, Wang J, Wei H, Wu X, Wu Y, Zhang S, Zhao S, Zhu Q, Cheatham TE 3rd, Roe DR, Roitberg A, Simmerling C, York DM, Nagan MC, Merz KMJ. 2023. AmberTools. J Chem Inf Model 63:6183–6191.

2.	Ruggiero M, Kerff F, Herman R, Sapunaric F, Galleni M, Gutkind G, Charlier P, Sauvage E, Power P. 2014. Crystal structure of the extended-spectrum β-lactamase PER-2 and insights into the role of specific residues in the interaction with β-lactams and β-lactamase inhibitors. Antimicrob Agents Chemother 58:5994–6002.

3.	Maier JA, Martinez C, Kasavajhala K, Wickstrom L, Hauser KE, Simmerling C. 2015. ff14SB: Improving the Accuracy of Protein Side Chain and Backbone Parameters from ff99SB. J Chem Theory Comput 11:3696–3713.

4.	Santos-Martins D, Solis-Vasquez L, Tillack AF, Sanner MF, Koch A, Forli S. 2021. Accelerating AutoDock4 with GPUs and Gradient-Based Local Search. J Chem Theory Comput 17:1060–1073.

5.	Frisch MJ, Trucks GW, Schlegel HB, Scuseria GE, Robb M a., Cheeseman JR, Scalmani G, Barone V, Petersson G a., Nakatsuji H, Li X, Caricato M, Marenich a. V., Bloino J, Janesko BG, Gomperts R, Mennucci B, Hratchian HP, Ortiz J V., Izmaylov a. F, Sonnenberg JL, Williams, Ding F, Lipparini F, Egidi F, Goings J, Peng B, Petrone A, Henderson T, Ranasinghe D, Zakrzewski VG, Gao J, Rega N, Zheng G, Liang W, Hada M, Ehara M, Toyota K, Fukuda R, Hasegawa J, Ishida M, Nakajima T, Honda Y, Kitao O, Nakai H, Vreven T, Throssell K, Montgomery Jr. J a., Peralta JE, Ogliaro F, Bearpark MJ, Heyd JJ, Brothers EN, Kudin KN, Staroverov VN, Keith T a., Kobayashi R, Normand J, Raghavachari K, Rendell a. P, Burant JC, Iyengar SS, Tomasi J, Cossi M, Millam JM, Klene M, Adamo C, Cammi R, Ochterski JW, Martin RL, Morokuma K, Farkas O, Foresman JB, Fox DJ. 2016. Gaussian 16, Revision C.01.
